# Microalgae colonization of different microplastic polymers in experimental mesocosms across an environmental gradient

**DOI:** 10.1111/gcb.15989

**Published:** 2021-12-03

**Authors:** Veronica Nava, Miguel G. Matias, Andreu Castillo‐Escrivà, Beata Messyasz, Barbara Leoni

**Affiliations:** ^1^ Department of Earth and Environmental Sciences University of Milano‐Bicocca Milano Italy; ^2^ Department of Biogeografía y Cambio Global Museo Nacional de Ciencias Naturales CSIC Madrid Spain; ^3^ MED—Mediterranean Institute for Agriculture, Environment and Development Rui Nabeiro Biodiversity Chair Universidade de Évora Évora Portugal; ^4^ Department of Hydrobiology Institute of Environmental Biology Adam Mickiewicz University in Poznan Poznań Poland

**Keywords:** biofouling, epiplastic community, periphyton, phytobenthos, plastic colonization, plastisphere

## Abstract

A variety of organisms can colonize microplastic surfaces through biofouling processes. Heterotrophic bacteria tend to be the focus of plastisphere research; however, the presence of epiplastic microalgae within the biofilm has been repeatedly documented. Despite the relevance of biofouling in determining the fate and effects of microplastics in aquatic systems, data about this process are still scarce, especially for freshwater ecosystems. Here, our goal was to evaluate the biomass development and species composition of biofilms on different plastic polymers and to investigate whether plastic substrates exert a strong enough selection to drive species sorting, overcoming other niche‐defining factors. We added microplastic pellets of high‐density polyethylene (HDPE), polyethylene terephthalate (PET), and a mix of the two polymers in 15 lentic mesocosms in five different locations of the Iberian Peninsula, and after one month, we evaluated species composition and biomass of microalgae developed on plastic surfaces. Our results, based on 45 samples, showed that colonization of plastic surfaces occurred in a range of lentic ecosystems covering a wide geographical gradient and different environmental conditions (e.g., nutrient concentration, conductivity, macrophyte coverage). We highlighted that total biomass differed based on the polymer considered, with higher biomass developed on PET substrate compared to HDPE. Microplastics supported the growth of a rich and diversified community of microalgae (242 species), with some cosmopolite species. However, we did not observe species‐specificity in the colonization of the different plastic polymers. Local species pool and nutrient concentration rather than polymeric composition seemed to be the determinant factor defying the community diversity. Regardless of specific environmental conditions, we showed that many species could coexist on the surface of relatively small plastic items, highlighting how microplastics may have considerable carrying capacity, with possible consequences on the wider ecological context.

## Graphical abstract

We performed a mesocosm experiment to study biomass development and species composition of microalgae on different plastic polymers. Our goal was to investigate whether plastic substrates exert a strong enough selection to drive species sorting, overcoming other niche‐defining factors. Colonization of plastic surfaces occurred in a range of lentic ecosystems covering a wide geographical gradient and different environmental conditions and we observed a rich and diversified community of microalgae. We did not observe species‐specificity in the colonization of the different polymers. Local species pool and nutrient concentration rather than polymeric composition seemed to be the determinant factor defying the community diversity.

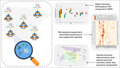

## 1 INTRODUCTION

While the benefits of plastics are undeniable, their widespread use as well as their inherent resistance to (bio)degradation ultimately leads to their accumulation in the environment (Thompson et al., 2009). In 2019, global plastic production almost reached 370 million tons and approximately 50% of plastic objects manufactured are intended for single‐use (Geyer et al., 2017; PlasticsEurope, 2020). As the production and use of plastic materials have intensified, the quantity of waste generated has also increased (Kedzierski et al., 2020). Just a small fraction of plastic waste is recycled (according to PlasticsEurope, 2020 the percentage worldwide was equal to 32.5% in 2018), while the remaining is incinerated or accumulates in landfills, eventually ending up in natural environments, including marine, and freshwater aquatic systems (Geyer et al., 2017). Previous studies estimated that ~8 million metric tons of plastic waste enter the ocean annually (Jambeck et al., 2015). Once in the aquatic environment, plastic debris undergoes mechanical, chemical, and biological modifications, which lead to the weathering and fragmentation of macroplastics into smaller and more abundant particles, forming the so‐called “microplastics” (MPs, <5 mm) (Julienne et al., 2019). Besides these degradation products (i.e., secondary microplastics), MPs can also be specifically manufactured within the millimetric size (i.e., primary MPs), like, for instance, those used as resin pellets or as an ingredient of personal care products (Horton et al., 2017). Beyond their effects as waste or pollutants, very little is known about the role of these particles when eventually colonized and incorporated as substrate by aquatic organisms.

Studies have shown that a wide variety of organisms can colonize microplastic surfaces through biofouling processes. Indeed, floating plastics represent a new habitat for rafting organisms to the point that the term “plastisphere” was coined to define the diverse community of heterotrophs, autotrophs, predators, and symbionts growing on the surface of plastic debris (Zettler et al., 2013). Even if heterotrophic bacteria tend to be the focus of plastisphere research, the presence of epiplastic microalgae (i.e., algae growing on plastic surfaces) within the biofilm has been repeatedly documented (Carpenter & Smith, 1972; Yokota et al., 2017). While it has been shown that the communities differ between biofilms and the ambient environment, there is no consensus on whether biofilms differ between substrates (Rogers et al., 2020). Experimental studies have reported differences in the communities found on plastic surfaces compared to other inert substrates, like for instance glass, suggesting substrate‐driven selection (e.g., Oberbeckmann et al., 2014; Ogonowski et al., 2018). Additionally, several lines of evidence indicate that the colonization process can vary depending on the plastic polymer used (Lagarde et al., 2016; Vosshage et al., 2018; Zettler et al., 2013). However, different research reported opposite results and it has been hypothesized that many taxa may use plastic opportunistically as a niche but can also attach to other substrates (Oberbeckmann et al., 2016; Smith et al., 2021).

The adhesion of microalgae on microplastics increases the density of the colonized polymer and, consequently, affects the vertical fluxes of plastics (Long et al., 2015). Therefore, biofouling processes are of critical importance for the fate of microplastics in aquatic systems, influencing their distribution along the water column and determining whether a particle occupies a pelagic versus benthic transport route. Moreover, it is reported that biofouling processes can alter the polymer features, influencing their capability to adsorb/desorb pollutants from the environments, with consequences for the toxic effects exerted by MPs (Kalčíková et al., 2020; Wang et al., 2020). Additionally, the interaction between microplastics and microalgae may have effects at the ecosystem level, as it is argued that MPs may affect algal productivity. Indeed, plastic debris provides a growth matrix and better floating conditions for microalgae and therefore it has been hypothesized that plastic pollution can promote the development of microalgae, with consequent detrimental effects for aquatic ecosystems already disturbed by eutrophication processes (Zhang et al., 2020).

Local conditions and environmental factors (e.g., temperature, nutrient concentration, salinity) influence the community composition of biofilm, and thus these variables play an important role in determining the development and diversity of MP‐colonizing communities (Amaral‐Zettler et al., 2015; Oberbeckmann et al., 2014). Indeed, the environmental context influences the development of periphyton, both with regard to their architectural structure and their taxonomic diversity and function (Villeneuve et al., 2010). However, there is still controversy as to whether substrate‐specific properties or environmental factors prevail in shaping microalgal assemblages on plastic debris. Indeed, it is not clear if the plastic surface “environment” may exert a strong enough selection to drive species sorting, overcoming other niche‐defining factors driven by seasonal and spatial patterns (Nava & Leoni, 2021).

Despite the importance of biofouling for microplastics in aquatic systems, data about this process are still scarce, especially for freshwater ecosystems, and mixed in terms of results due to different confounding factors that arise from in‐field experiments. To better understand the process of colonization by microalgae of different microplastic polymers and to identify possible shaping factors, we performed an experiment using a multi‐site mesocosm experimental system distributed across an environmental gradient in five different locations of the Iberian Peninsula (Spain, Portugal). These systems are characterized by a naturalized microalgae community with over 4 years of colonization. In each site, we deployed microplastic pellets of high‐density polyethylene (a floating plastic), polyethylene terephthalate (a polymer denser than water), or a mix of the two polymers. Following one month of colonization, we determined species composition and quantified biomass of microalgae developed on plastic pellets. The resulting dataset allowed us to: (i) evaluate whether different plastic polymers constituted suitable substrates for the development of microalgal communities; (ii) quantify the microalgae biomass developed on microplastics with different density and polymeric composition and determine whether biomass vary significantly among substrates; (iii) identify which algal species were able to colonize different plastic polymers; (iv) determine whether substrate‐driven or environmental factors prevail in shaping the species diversity of epiplastic community.

## 2 METHODS

### 2.1 Study area

**FIGURE 1:**
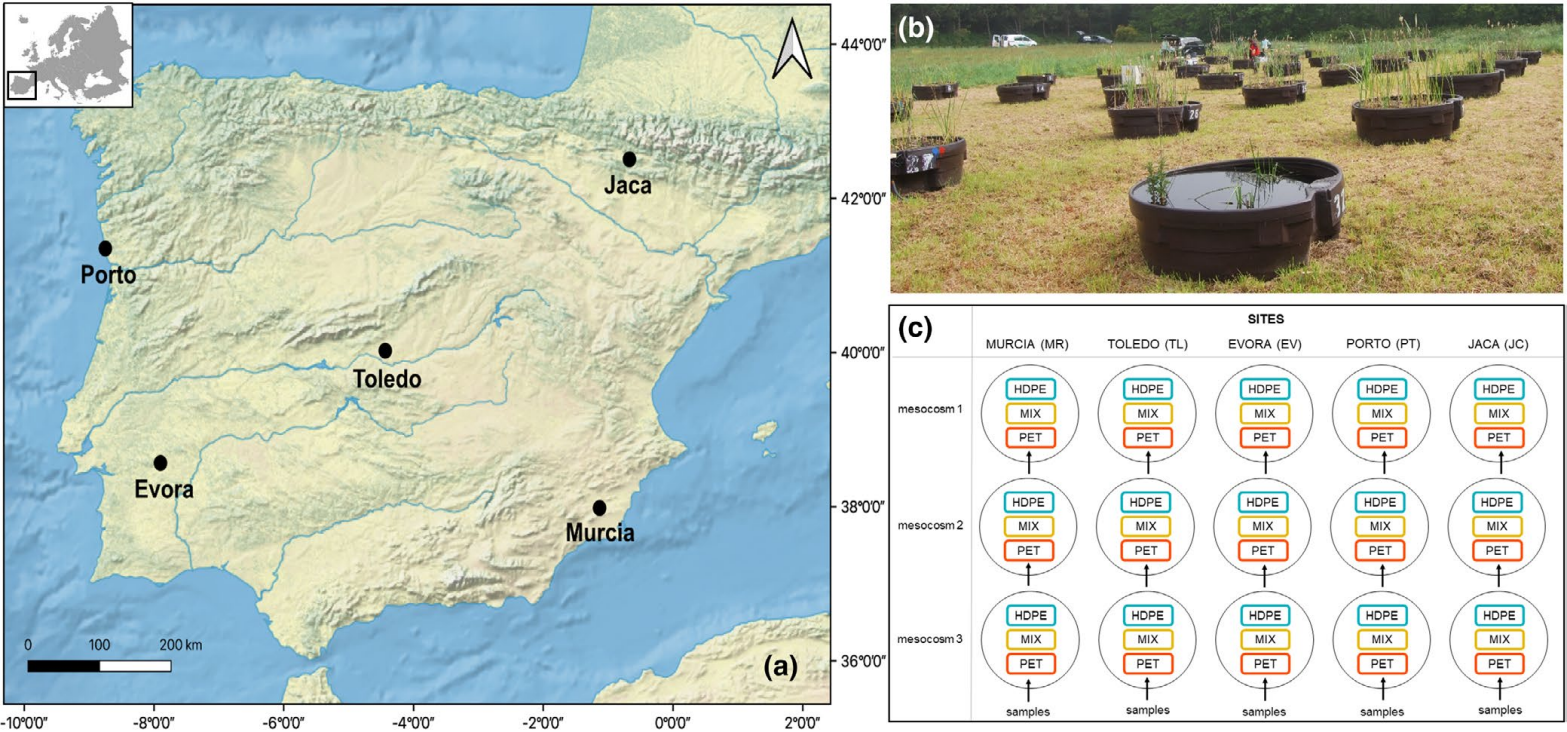
(a) Study area with location of the sites in which mesocosms are deployed. (b) Example picture of freshwater mesocosms (1000 L tanks). (c) Schematic representation of the 5 sites selected for our experiment, the 15 mesocosms (with three enclosures each), and the resulting 45 samples (of which 15 with HDPE, 15 with MIX, and 15 with PET)

The Iberian Pond Network (IPN, https://www.aquacosm.eu/mesocosm/iberian‐pond‐network‐ipn/, Pereira et al., 2021) is a multi‐region mesocosm system built in 2014 to investigate ecological responses to climate change and anthropogenic impacts across bioclimatic regions (Figure 1a). This infrastructure includes a range of environments from semi‐arid conditions to mountain tops. A total of 192 mesocosms, each 0.70 m deep and 1.85 m in diameter, are deployed across 6 regions on the Iberian Peninsula. Locations include semi‐arid, Mediterranean, temperate, and alpine environments (Pereira et al., 2021). At each location, 32 mesocosms with a volume of 1000 L each are placed at ~3–5 m distance from one another (Figure 1b). The mesocosms were initiated by adding 100 kg of locally collected topsoil, then filled with local water. All mesocosms have been left untouched until 2019 to allow the establishment of aquatic food webs. For the present study, we selected five sites (i.e., Murcia, “MR”; Toledo, “TL”; Evora, “EV”; Porto, “PT”; Jaca, “JC”) and three mesocosms for each site; furthermore, three enclosures were deployed in each mesocosm (Figure 1c).

Murcia has a Mediterranean climate with semi‐arid features. The average annual temperature ranges from 15.0°C to 19.0°C and the annual rainfall is less than 350 mm (Alonso‐Sarría et al., 2016). The climate in Toledo is continental semiarid with an annual rainfall of 487 mm and an average annual temperature of 14.0°C (Hernández et al., 2007). Evora has a typical Mediterranean climate, with hot and dry summers. More than 80% of annual precipitation occurs between October and April. The long‐term mean annual temperature is 15.0–16.0°C, with 669 mm of precipitation on average (Pereira et al., 2007). Due to the maritime influence, Porto has mild temperatures with an annual average of 14.4°C. No cold season can be found in Porto, with January being the coldest month, with an average temperature of 9.3°C. The mean summer temperature is about 18.1°C, although very high temperatures can be reached between May and September. The most significant feature of the Porto climate is the annual rainfall level (1236 mm) which has an irregular distribution throughout the year, mainly concentrated in winter and spring (Abreu et al., 2003). In Jaca, climate conditions are typically alpine, with average annual temperatures that range between −0.7°C and 5.0°C and high‐mean annual precipitation values well distributed throughout the year (Garcia‐Pausas et al., 2007).

### 2.2 Field experiment

The experiment was carried out in spring and summer 2019. We employed virgin plastic pellets, provided by Serioplast Global Services. We used high‐density polyethylene (HDPE) and polyethylene terephthalate (PET), which are among the polymers more commonly found in freshwater systems (Li et al., 2020). Before use, plastic polymers were characterized through micro‐Raman spectroscopy (Raman Horiba Jobin Yvon LabRAM HR Evolution; Nava et al., 2021). The Raman system is equipped with an Nd laser (532 nm) and a cooled charge‐coupled device (CCD, 1024 × 256 px, −60°C) detector. A grating with 600 grooves/µm was used. Raman spectra were recorded with a 50× objective (Olympus BXFM) with an integration time of 30 seconds. The spectral range was set to 223–3177 cm^−1^ and the spectral resolution was equal to 1.47 counts/points (Figure S1).

To prevent contamination of the ongoing experiments in the mesocosms, smaller enclosures were prepared. These consisted of containers with two openings of 15 × 5 cm (75 cm^2^ area), covered by a net with a mesh size of 100 µm. Three experimental treatments were implemented in each mesocosm: 6 g of high‐density polyethylene (HDPE), 6 g of polyethylene terephthalate (PET) or a mixture of the two polymers (MIX, 3 g each). Overall, we evaluated 5 locations, 15 mesocosms, and 45 samples, of which 15 HDPE, 15 PET, and 15 MIX (Figure 1c).

Several physical and chemical parameters were measured in each mesocosm. Water temperature, pH, turbidity, and conductivity were measured using a multi‐parameter probe (Hach HQ40D). Chlorophyll‐a was measured using an AquaFluor (Turner Designs) portable fluorometer. Nutrients (i.e., nitrate, NO_3_^−^; ammonium, NH_4_^+^; phosphate, PO_4_^3−^; silicate, SiO_4_^4−^) were measured following standard methods (APHA/AWWA/WEF, 2012). After one month, microplastics from each site and mesocosm were collected and placed in a 50 mL sterile Falcon tube with a known volume of mesocosm water (filtered before use); then, plastic pellets were scraped off using a small sterile conical brush. Then, samples have been gently mixed and preserved in Lugol solution. Before the identification in the laboratory, microplastics were visually inspected under an optical microscope to ensure that the surfaces were properly cleaned, and no remaining microalgae were left attached to the polymer surfaces.

### 2.3 Laboratory analyses

Algae were counted and identified with an optical microscope at 400X magnifications in a Utermöhl chamber following the Utermöhl technique (EN 15204:2006). To identify diatoms, permanent slides were prepared using standard procedures: the samples were heated with 30% hydrogen peroxide (H_2_O_2_) for at least four hours to oxidize organic material, then we added concentrated hydrochloride acid (HCl, 1 M) to remove carbonates and finally we rinsed the processed material with distilled water in four centrifugation steps (Battarbee, 1986). Cleaned material was transferred on a 24 × 24 mm coverslip and a drop of Naphrax (R.I. = 1.7) was used to mount the slides. Diatoms were identified with the optical microscope with 1000× magnification under oil immersion. Identification of microalgal species was based on the microscopic analysis of their morphological features, according to specific identification keys (Komárek & Anagnostidis, 2007; Lange‐Bertalot et al., 2017), updated to recent taxonomic nomenclature using Internet databases (Gury & Gury, 2021). The biovolume of each species was determined through a volumetric analysis of cells using geometric approximation and expressed as a weight following Wetzel and Likens (2000). Algal density (cell cm^−2^) and biomass (µg cm^−2^) were estimated.

### 2.4 Data analyses

Samples collected from the three mesocosms within the same site and with the same treatment of plastic polymer were considered as replicates (Figure 1c). Relationships between biomass across the different samples and sites have been evaluated through linear mixed‐effect models (LME, Zuur et al., 2009). As fixed effects, we entered site and plastic‐type (with interaction term) into the model. As random effects, we included the different mesocosms nested in the sites. *p* values were obtained by F test on fixed effects using Satterthwaite approximation.

Alpha diversity (i.e., the number of taxa or number of functional characteristics within a location, cf. Rolls et al., 2018) was evaluated using the Shannon index, the inverse of Simpson index, and Pielou evenness index. To measure the association between species and the different levels tested (i.e., polymer type and site), we used the composite index called “IndVal” (indicator value) by Dufrêne and Legendre (1997), which ranges from 0, no association, to 1, maximum association. Significant differences in sample diversity were assessed through Kruskal–Wallis test.

Differences in microalgal communities among samples (Beta diversity, cf. Rolls et al., 2018) were analyzed by non‐metric multidimensional scaling (NMDS), based on Bray & Curtis’ dissimilarity distances (Legendre & Legendre, 1998) calculated from the biomass of the different species. Before NMDS computation, the data were transformed by double square root to reduce the importance of the more abundant taxa (Salmaso, 2010). As an indicator of fitness, a stress function that measures the fit between NMDS distance and actual dissimilarities was calculated. A stress value (STR) > 0.20 provides a representation not different from random, STR < 0.15 a good representation, and STR < 0.10 an ideal representation (Clarke, 1993). The significance of main effects (based on “site” and “polymer type” groups) was tested using permutational multivariate analyses of variances (PERMANOVA) applied to the distance matrix used as input for the NMDS ordination of samples with 999 permutations. Physical and chemical variables were related to the strongest gradients in species composition by fitting environmental vectors to the NMDS configurations. The significance of vectors was based on 999 random permutations of the data. To further evaluate the type of relationship between configurations and environment, a few selected variables were related to the gradients in species composition by surface fitting. All the analyses were carried out in R 4.0.3 (R Core Team, 2020), using the following packages: “lmerTest” (Kuznetsova et al., 2017), “vegan” (Oksanen et al., 2012), and “ggplot2” (Wickham, 2016).

## 3 RESULTS

### 3.1 Physical and chemical parameters

Mesocosm waters over the five locations differ based on meteorological data and chemical and physical parameters (Table 1 and Table S1). Mean air temperature, over the experimental period, ranged between 17.0 and 21.0°C across the different locations, while cumulative rainfall varied between 0 and 115 mm (Table S1). High values of electrical conductivity (EC) were recorded in Murcia, as a result of dry conditions and small rainfall amount, reaching values wide above the usual range in freshwater systems. Slightly acid conditions were highlighted in Porto, while in the remaining locations pH was almost neutral (Jaca, Evora) or alkaline (Toledo, Murcia). Phosphate (PO_4_^3−^) reached high values in Toledo and, especially, in Evora, with a concentration above 3 mg L^−1^; differently, Murcia, Porto, and Jaca showed lower concentration with values spanning from 3 to 50 µg L^−1^. Nitrate (NO_3_^−^) concentrations were quite similar across locations, ranging from values around 0.003 ± 0.003 mg L^−1^ in Toledo to a concentration of 0.17 ± 0.02 mg L^−1^ in Jaca. Ammonium ranges from 0.2 to 0.7 mg L^−1^ in all the locations, except for Toledo where the concentration reached 2.25 ± 0.84 mg L^−1^. The highest values of silicate (SiO_4_^4−^) were highlighted in Toledo and Evora, even if with marked intra‐site differences. Macrophytes are absent in Toledo and low coverage was observed in Jaca (~38%); instead, a mean coverage above 60% was recorded in Porto (~63%, *Typha* sp.), Evora (~88%, *Typha* sp. and *Lemna* sp.) and especially in Murcia, with almost full coverage (~99%) dominated by *Zannichellia* sp.

**TABLE 1:** Mean (±standard error) of physical and chemical parameters measured in the three mesocosms selected for each site

	Murcia (MR)	Toledo (TL)	Evora (EV)	Porto (PT)	Jaca (JC)
Water temperature (°C)	19.0 ± 0.4	9.3 ± 0.6	15.7 ± 0.5	15.5 ± 0.1	15.5 ± 0.6
EC (µS cm^−1^)	22 046 ± 460	2840 ± 46	893 ± 109	69 ± 16	276 ± 45
Turbidity (NTU)	2.19 ± 1.67	27.35 ± 21.21	8.88 ± 1.33	3.37 ± 0.77	3.27 ± 0.84
pH	10.37 ± 0.13	9.78 ± 0.40	7.91 ± 0.32	6.40 ± 0.26	7.73 ± 0.16
NO_3_^−^ (mg L^−1^)	0.097 ± 0.031	0.003 ± 0.003	0.134 ± 0.035	0.012 ± 0.003	0.170 ± 0.023
PO_4_^3−^ (mg L^−1^)	0.046 ± 0.044	1.960 ± 1.168	3.219 ± 0.918	0.003 ± 0.003	0.016 ± 0.013
NH_4_^+^ (mg L^−1^)	0.706 ± 0.078	2.253 ± 0.836	0.625 ± 0.086	0.194 ± 0.061	0.245 ± 0.058
SiO_4_^4−^ (mg L^−1^)	4.777 ± 3.099	62.207 ± 11.940	17.277 ± 14.787	1.134 ± 0.333	5.379 ± 4.955
Macrophyte coverage (%)	99 ± 1	0 ± 0	88 ± 13	63 ± 7	38 ± 22

### 3.2 Density and biomass distribution

**FIGURE 2:**
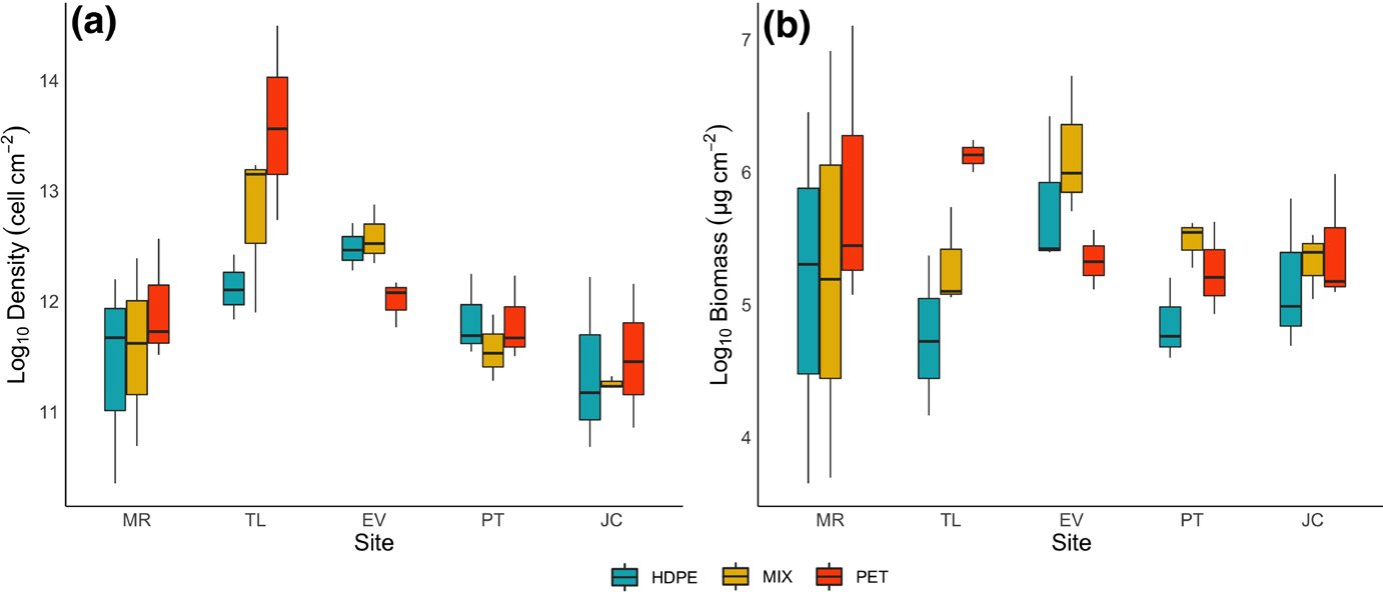
Tukey boxplot of (a) density (cell cm^−2^), and (b) biomass (µg cm^−2^) of microalgae for different sites on the two plastic polymers and the “MIX” treatment. MR, Murcia; TL, Toledo; EV, Evora; PT, Porto; JC, Jaca

Regardless of sites or plastic polymers, all the 45 samples analyzed have been colonized by microalgae. The mean microalgal density developed on HDPE substrate was equal to 1.6 × 10^5^ ± 2.2 × 10^4^ (mean ± standard error) cell cm^−2^, presenting on average less pronounced colonization compared to MIX, where the mean density was of 2.0 × 10^5^ ± 4.2 × 10^4^ cell cm^−2^, and especially to PET with a value of 3.2 × 10^5^ ± 1.3 × 10^5^ cell cm^−2^ (Figure 2a). Considering the different sites, the highest density has been highlighted in Toledo with a mean value of 5.4 × 10^5^ ± 1.9 × 10^5^ cell cm^−2^, followed by Evora (2.4 × 10^5^ ± 2.7 × 10^4^ cell cm^−2^), Murcia (1.4 × 10^5^ ± 2.9 × 10^4^ cell cm^−2^), Porto (1.3 × 10^5^ ± 1.5 × 10^4^ cell cm^−2^), and Jaca (9.8 × 10^4^ ± 1.9 × 10^4^ cell cm^−2^; Figure 2a).

Considering the biomass values, the average value for the samples collected on HDPE substrate is 219.7 ± 46.4 µg cm^−2^, while the average value for MIX and PET is equal to 315.1 ± 67.3 µg cm^−2^ and 329.1 ± 70.5 µg cm^−2^, respectively (Figure 2b). Biomass values across the several locations are equal to 410.8 ± 145.3 µg cm^−2^ in Murcia, 357.5 ± 74.7 µg cm^−2^ in Evora, 265.4 ± 53.8 µg cm^−2^ in Toledo, 215.6 ± 31.5 µg cm^−2^ in Jaca, and 190.5 ± 22.2 µg cm^−2^ in Porto (Figure 2b).

Generally, within the sites, higher values of biomass were detected on PET compared to HDPE. This is verified for all the sites except for Evora, in which, in contrast, PET samples showed the lowest microalgal biomass. This difference observed in Evora was mainly linked to one mesocosm (i.e., EV‐2), in which the biomass developed on PET substrate was much lower than the biomass on HDPE (Figure S2). Biomass developed on MIX samples is generally higher than the biomass of HDPE samples; the evidence, however, is controversial when comparing results of MIX with PET samples, since for Murcia and Toledo mean biomass on MIX is lower than PET, while for the Evora, Porto, and Jaca the opposite is true.

Results of the linear mixed‐effect model, reported in Table 2a, indicate that there are significant differences in total biomass colonizing different plastic types (*p* < .05), although the magnitude of these differences varies across sites (“plastic × site” interaction; *p* < .05), while the site is not a significant factor (Table 2a).

**TABLE 2:** Results of linear mixed‐effect model (LME) testing effect of plastic type (“plastic,” three levels: HDPE, MIX, and PET), site (“site,” five levels: MR, TL, EV, PT, and JC) and their interaction (“plastic × site”) on (a) total biomass; (b) biomass of different taxa

	Plastic			Site			Plastic × site		
	df	F	*p*	df	F	*p*	df	F	*p*
(a)									
Total biomass	2	3.881	.038*	4	0.514	.727	8	2.886	.026*
(b)									
Diatom biomass	2	4.049	.028*	4	2.467	.113	—	—	—
Ochrophyta biomass	2	1.911	.174	4	8.091	.003**	8	2.988	.022*

Note

Bold indicates significant values.

Significant value: *p < .05; **p < .01; ***p < .001.

Across all samples, the taxa that gave the major contribution to total biomass were Bacillariophyta (diatoms) and Miozoa (Dinophyceae), with an average relative abundance of 28.7 ± 3.2% and 27.5 ± 3.4% (mean ± standard error), respectively. Considering the different polymers, HDPE had on average a higher abundance of diatoms, with a mean value of 59.2 ± 13.5 µg cm^−2^; instead, Miozoa was the taxon with the greatest abundance in MIX and PET samples (107.7 ± 40.5 µg cm^−2^ for MIX; 111.0 ± 54.0 µg cm^−2^ for PET; Figure S3). Porto and Jaca were mainly dominated by Miozoa, representing on average 37.8 ± 6.4% and 50.6 ± 3.4% of the biomass, respectively. Bacillariophyta provided the major contribution to microalgae biomass in Evora (46.4 ± 8.1%) and Murcia (47.7 ± 5.0%); while in Toledo, Ochrophyta represented 40.1 ± 5.2% of the biomass (Figure S3).

Significant differences of the linear mixed model performed on the biomass of the different taxa (phyla = 9) were only found for Bacillariophyta and Ochrophyta (Table 2b). For Bacillariophyta, significant differences were found for different polymers (*p* < .05), with higher biomass of diatoms being found on PET samples when compared to HDPE. For Ochrophyta, significant differences in biomass were found only among sites (*p* < .01), and the post hoc pairwise comparison highlighted that the site that significantly differed from the others is Toledo, where we observed a high abundance of microalgae belonging to this phylum.

### 3.3 Alpha diversity and community composition

**FIGURE 3:**
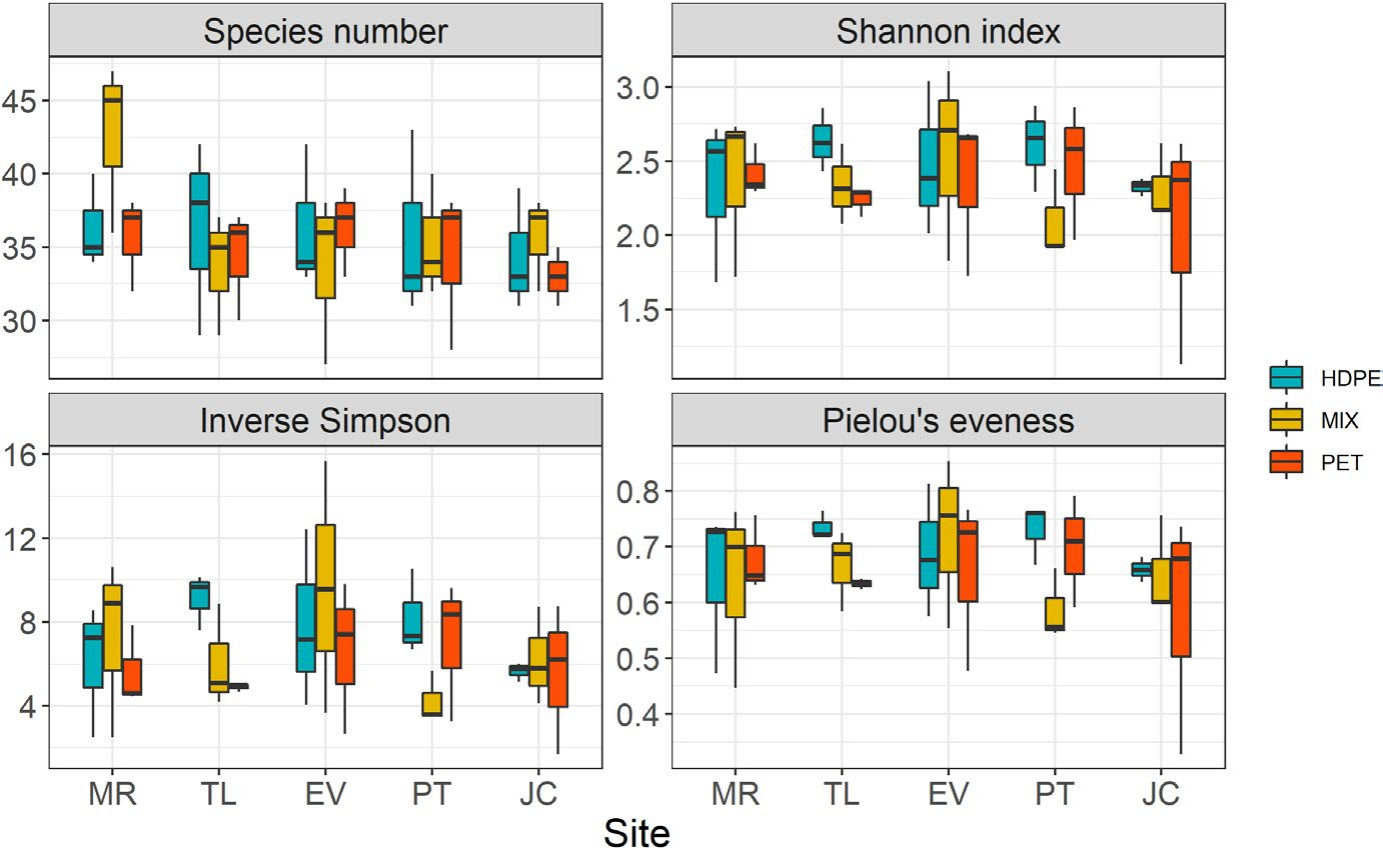
Alpha‐diversity for different plastic types expressed as number of species (“Species number”), Shannon index (“Shannon index”), inverse of Simpson index (“Inverse Simpson”), and Pielou's evenness index (“Pielou's evenness”)

The average number of species among all samples is equal to 35 ± 1, with a maximum value of 47 (identified in the MIX sample in Evora) and a minimum of 27 (highlighted in the MIX sample in Murcia). Considering the different polymers, alpha diversity (expressed by the Shannon index, inverse Simpson index, and Pielou's evenness) displayed slightly higher values on HDPE compared to PET samples; however, these differences are not remarkable, and no significant differences were highlighted by Kruskal–Wallis test among groups (Figure 3).

Over the 45 samples analyzed, we found 242 different species distributed in 144 genera. The 33.5% belong to the phylum Bacillariophyta, followed by 26.4% Chlorophyta, 11.6% Cyanobacteria, 8.3% Charophyta, 6.6% Euglenozoa, 6.6% Ochrophyta, 4.1% Miozoa, 2.5% Cryptophyta, and 0.4% Haptophyta. We found some cosmopolite species, which were recorded in almost all the samples, like *Aphanocapsa incerta* (Lemm.) Cronberg & Komárek identified in 44 over 45 samples. Besides this, the most frequent species were *Cocconeis placentula* Ehr. identified in 39 samples; *Peridiniopsis elpatiewskyi* (Ostenf.) Bourrelly in 37 samples; *Achnanthidium minutissimum* (Kütz.) Czarnecki in 36 samples; *Cocconeis pediculus* Ehr. in 35 samples; *Planktolyngbya limnetica* (Lemm.) Kom.‐Legn. & Cronberg in 33 samples. A total of 48 species were present only in one sample and 41 in just two samples. Overall, 20 species from 19 genera, with a mean relative abundance of 1.4 ± 0.1%, occurred only in HDPE samples, and 17 species from 16 genera only in PET samples, with an average relative abundance of 0.9 ± 0.1%. However, in all the cases these species had very low recurrence, being identified at most in four different samples.

**FIGURE 4:**
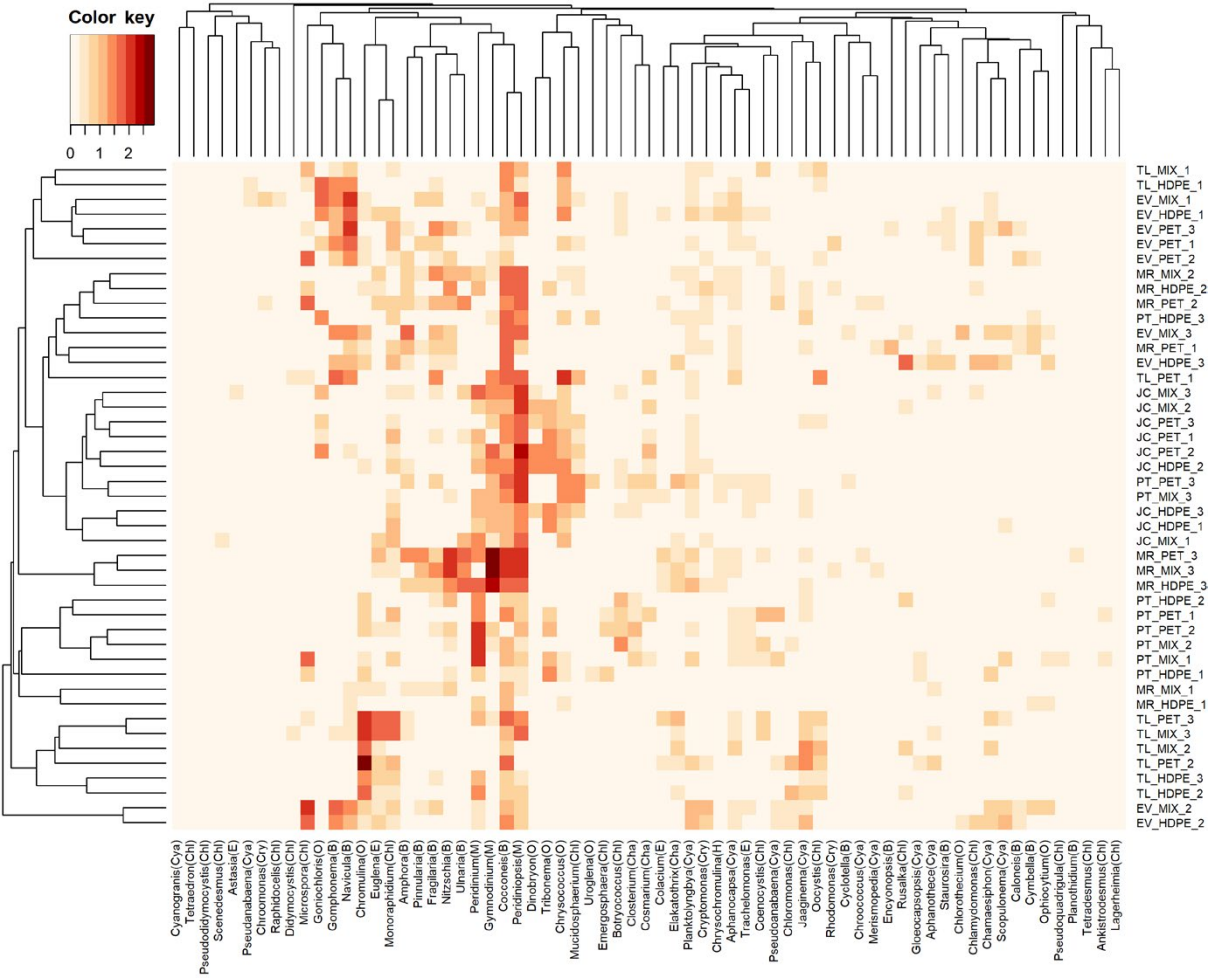
Heatmap visualizing the biomass distribution of genera of microalgae across the samples. Only genera that were identified in at least 10% of the samples (*n* = 5) are reported. Biomass data were log_10_(x + 1) transformed before plotting. Clusters have been calculated based on Bray‐Curtis distance. Clusters of samples (row cluster) have been calculated considering all the genera identified. The corresponding phylum for each genus is given in brackets: “Cya” Cyanobacteria, “B” Bacillariophyta, “Chl” Chlorophyta, “Cha” Charophyta, “E” Euglenozoa, “O” Ochrophyta, “Cry” Cryptophyta, and “M” Miozoa

The biomass distribution of the most recurrent genera across the samples is reported in Figure 4. The highest contribution to biomass was provided by the following species: *Gymnodinium discoidale* Harris (33.3 ± 20.0 µg cm^−2^); *Peridiniopsis cunningtonii* Lemm. (22.8 ± 5.9 µg cm^−2^); *Dictyosphaerium ehrenbergianum* Nägeli (20.1 ± 14.0 µg cm^−2^); *Chromulina pseudonebulosa* Pascher (18.1 ± 9.3 µg cm^−2^); *Cocconeis pediculus* (17.1 ± 3.0 µg cm^−2^). In particular, the genera *Cocconeis* and *Peridiniopsis*, besides having a high abundance, also had a high recurrence. The most species‐rich genera were *Gomphonema* and *Navicula*, with a maximum of 7 and 6 taxa, respectively.

### 3.4 Beta diversity and relationship with environmental variables

Cluster analysis based on the Bray‐Curtis similarity index calculated on community composition (Figure 4) did not discriminate samples based on the different polymers colonized (HDPE, PET or MIX), but rather by the different sites, which seems to be a more influential factor affecting community composition. This is confirmed by the IndVal index since no significant indicator species were identified based on the different polymers. Indicator species were in turn highlighted for the different sites, showing the highest values (*p* = .001) for the genera *Nitzschia*, *Pinnularia*, *Ulnaria*, and *Merismopedia* in Murcia, *Oocystis* in Toledo, *Navicula*, *Caloneis*, *Staurosira*, and *Gomphonema* in Evora, *Closterium* in Porto, and *Dinobryon* in Jaca (Table S2).

**FIGURE 5:**
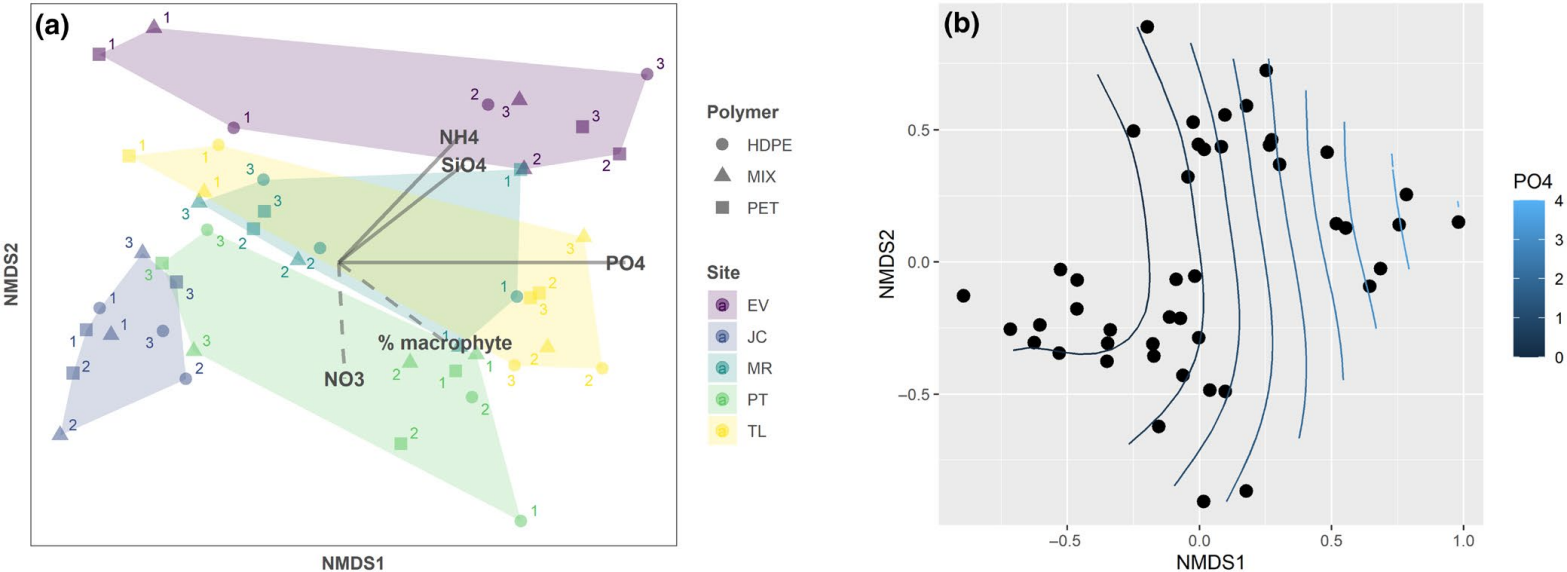
(a) Non‐metric multidimensional scaling (NMDS) run on species‐level microalgae biomass with vectors of significant environmental variables. Vectors are significant at *p* < .05 (solid arrows) and *p* < .10 (dashed arrows). “PO4”: phosphate concentration; “NH4”: ammonium concentration; “SiO4”: silicate concentration; “NO3”: nitrate concentration; “% macrophyte”: coverage of macrophyte in percentage. (b) Surface fitting for the PO_4_^3−^ concentration (bin width = 0.5)

The ordination of the samples through NMDS analysis (three‐dimensional solution, STR = 0.14) based on the species‐specific biomass composition allowed separating the samples mostly based on the geographic position (Figure 5a). In particular, a noticeable difference in community composition is evident for samples collected in Jaca, with a clear separation compared to the other sites. The separation of the groups based on the geographic position was confirmed by the PERMANOVA analysis (*p* = .001). Significant differences were not highlighted, instead, considering the polymer colonized.

The variation in species composition along the first axis was positively linked to phosphate (PO_4_^3−^) concentration, which had the highest importance in the ordination space. This result suggests that phosphate concentration may be the dominant driver of the microalgae community composition. The high association of species composition with the gradient of phosphate concentration is further illustrated by the pattern of PO_4_^3−^ in the NMDS configuration (Figure 5b). Other significant environmental variables included silicate (SiO_4_^4−^) and ammonium (NH_4_^+^), which also contributed to explaining the variation along the second NMDS axis. The macrophyte coverage (% macrophyte) was associated with both the first and the second axis, but its importance is also limited. Instead, conductivity and pH were not significant variables.

## 4 DISCUSSION

In a mesocosm investigation across a geographical gradient, this study has highlighted that different microplastic polymers represent a substrate that can be widely colonized by a diverse community of microalgae. Indeed, biofouling of the surfaces of HDPE and PET microplastics occurred in all conditions, regardless of the sites and the plastic polymer considered. This is in line with the available literature in which it is reported that microplastics provide new niches in the aquatic environment and, thus, represent available and long‐lasting substrates for a diverse microbial community (Rummel et al., 2017; Zettler et al., 2013).

Our results showed that colonization occurred in a range of lentic ecosystems since mesocosms used for our experiment cover a wide geographical gradient and different environmental conditions. Colonization of plastic surfaces by microorganisms has been reported for microplastics collected in a variety of aquatic systems (e.g., Debroas et al., 2017; Dussud et al., 2018; Oberbeckmann et al., 2014). However, to date, studies in freshwater systems are numerically less abundant than the ones in marine environments, and there is a need to increase the knowledge of plastisphere consortia in freshwater systems (Harrison et al., 2018). Besides, the majority of the studies have focused on the bacterial community, neglecting or marginally considering microalgae community in epiplastic assemblages, which are fundamental components at the base of aquatic food webs and pivotal organisms in a broad variety of ecosystem functions (Nava & Leoni, 2021). In our studies, we performed a thorough microscopic (phenotypic) investigation of the microalgae community in the epiplastic assemblage, which allowed evaluating the community diversity and the abundance of the different microalgae species. We highlighted that total biomass differed based on the polymer considered, with higher biomass developed on PET substrate compared to HDPE. This result seemed to be linked especially to diatoms since the outcome of the linear mixed model highlighted differences of total biomass only for this taxon, which constitute one of the most diverse and numerically abundant groups. The different amounts of biomass developed on the two polymers may be linked to the differential position of HDPE (i.e., floating) and PET (i.e., sinking) on the water column. Indeed, it is possible that microalgae community developed on floating plastics were more exposed to UV radiation with subsequent photoinhibition effect, which could have limited algae growth, or physical abrasion of biofilm of floating particles could have occurred (Arias‐Andres et al., 2018; Raven & Waite, 2004).

It seems now clear that microbial communities on plastic debris differ consistently from the surrounding aquatic communities, as the presence of an additional substrate constitutes a new niche with the possibility of development for a distinct community (Wright et al., 2020; Yang et al., 2020). The discussion is now moved toward whether different substrates could allow the growth of distinct species. Our results highlighted a rich and diversified community of microalgae developed on both HDPE and PET substrate, but we did not observe species‐specificity in the colonization of the different plastic polymers. Indeed, local species pool rather than polymeric composition seems to be the determinant factor defying the community diversity. We hypothesize that the existing communities in the different mesocosms may be responsible for some of the trends in species assemblages and future studies should address this relationship. Indeed, previous research, investigating periphyton assemblages in temperate lakes, reported as many of the algae identified in the periphyton were common components of the phytoplankton community that had likely settled out of the water column (Wood et al., 2012) and other studies have also shown that regional microalgae species richness have a strong influence on the richness of periphyton algae communities (Algarte et al., 2017). But still, additional factors contribute in determining species diversity of phytobenthos, like abiotic and biotic factors such as nutrient levels, temperature, light, and grazing, but also habitat heterogeneity, and hydrological factors (Algarte et al., 2017).

Previous research from marine environments showed similar results to those observed in this study, reporting that geography is likely to be a stronger predictor of plastisphere community composition at the scale of ocean basins (Harrison et al., 2018; Oberbeckmann et al., 2021). However, this is still under debate with several studies suggesting substrate‐driven selection, with differences reported not only when comparing inert control material to plastic substrates (e.g., Miao et al., 2019; Ogonowski et al., 2018) but also when colonization on different plastic polymers was evaluated (e.g., Li et al., 2019; Pinto et al., 2019). However, since the term plastics includes a plethora of different polymers, with different chemical and physical features and different additives, results should be extended and compared with caution (Yang et al., 2020). Another variable that should be taken into account is the study duration, whose variation may influence the process and the conclusions drawn (Nava & Leoni, 2021). Indeed, previous studies have shown that there are strong shifts and distinct communities during early stages of colonization. Over time, however, communities converge and remain stable in mature biofilms (Pinto et al., 2019; Wright et al., 2020). This may be explained as only the initial recruits have direct contact with the polymer surface; in contrast, later recruits are more likely to interact with existing biofilm members and the abiotic components of the surrounding environment (Dudek et al., 2020; Oberbeckmann et al., 2016). It is reported as stable and consistent epiplastic bacterial communities can be achieved within days to over one week, while the establishment of a mature eukaryotic community may take longer (Erni‐Cassola et al., 2020; Lobelle & Cunliffe, 2011). The one‐month duration for our study may have allowed observing a more developed microalgal community with different recruits not directly attached to the polymer surfaces and hence it could be hypothesized that this may constitute the cause of the absence of clear divergence in community composition on HDPE compared to PET. However, it is difficult to define the time frame in which the community becomes mature as this can also vary depending on different factors and environmental conditions. For instance, Smith et al. (2021), studying the evolution of algal biofilm assemblages on plastic polymers over time, reported significant differences between diatom assemblages also between week 4 and week 6, demonstrating as differences in community composition can be also observed in a longer period of colonization. At the same time, it is reported as differences between materials are usually driven by rare taxa (Pinto et al., 2019). We identified 17 species developed exclusively on PET and 20 species exclusively on HDPE with a low relative abundance. However, we cannot exclude stochastic processes determining the presence of distinct species on the different substrates.

Species belonging to the phylum Bacillariophyta were among the most abundant, and the most diverse in almost all the sites. Most studies have shown that diatoms are common and omnipresent residents of the plastisphere, at least on plastics that are exposed to sunlight (Amaral‐Zettler et al., 2020). Common diatoms reported in previous plastisphere research include species belonging, for instance, to genera *Cocconeis*, *Amphora*, *Fragilaria*, *Navicula*, and *Nitzschia* (e.g., Dudek et al., 2020; Oberbeckmann et al., 2014; Reisser et al., 2014), which were all observed in our study. Besides diatoms, Cyanobacteria and Chlorophyta were the only taxa for which we identified species in 100% of samples analyzed. Indeed, diatoms, cyanobacteria, and green algae have been reported as pioneering microbes that colonize plastic debris and their presence is also widely reported (Wright et al., 2020). However, it is difficult to compare results about community composition with previous studies since different “location‐specific” factors (i.e., microbial community, environmental condition, macrophyte coverage) determine the development and growth of the species on plastic surfaces (Yang et al., 2020). For instance, different macrophytes, which have different architecture and constitute an important parameter for periphytic algal community organization (Messyasz et al., 2009; dos Santos et al., 2013), might represent a source of microalgae species that can later develop on microplastic. However, our analyses showed that macrophyte coverage did not strongly affect community composition. Among the different environmental conditions, nutrient concentration has been reported as one of the most influential factors influencing microplastic biofilm structure (Li et al., 2019; Oberbeckmann et al., 2018). Our results are consistent with these findings, as we showed that nutrient concentration, and in particular phosphate concentration, was pivotal in determining the species assemblages on plastic surfaces, as highlighted by the NMDS analysis. In sites where the concentration of phosphate was low, like Porto and Jaca, we observed a slightly lower microalgae growth, even if we did not find any relationship between phosphate concentration and total biomass (see Figure S4); however, we identified a high number of species in Porto and Jaca, with almost identical values to those of sites and mesocosms with higher nutrient concentrations. This showed that the presence of microplastics, offering a new substrate on which microalgae can grow, may promote the development of a high number of diverse species of microalgae with values comparable to those observed in environments with higher nutrient concentrations. It has been already highlighted as floating plastics, which also increases the entrapment of nutrients from the surrounding environment, may constitute net autotrophic “hot spots” in the oligotrophic ocean, with a high density of chlorophyll and high oxygen production (Bryant et al., 2016; Chen et al., 2020). This may also happen in freshwater ecosystems with consistent development of epiplastic algae also in oligotrophic environments. Regardless of specific environmental conditions, we showed that many species can coexist on the surface of relatively small plastic items, highlighting as microplastics may have considerable carrying capacity, with possible consequences on the wider ecological context, for both aquatic food webs and ecosystem functioning (Nava & Leoni, 2021; Wright et al., 2020).

## 5 CONCLUSIONS

Outcomes of this study highlighted that microplastics represent an available substrate for the colonization by a variety of phytobenthic organisms in freshwater ecosystems. Small surfaces of plastics may host many different species of microalgae, but we did not observe a dissimilarity in community composition based on distinct polymeric composition, corroborating findings from the previous research conducted mainly in marine ecosystems. Local species pool and nutrient concentration seem to be the most crucial factors in driven species sorting of epiplastic community. Future studies should look at both the existing microalgal assemblages in water and the new assemblages that form on plastic polymer samples in order to understand the relationship between them.

Differences based on the polymer types were highlighted, instead, for the total biomass of microalgae, which was, however, high in all the samples in both eutrophic and oligotrophic systems. In a broader context, the considerable growth of primary producers on microplastic particles, whose presence is argued to be relevant in freshwater systems and expected to increase in the future, may have important consequences on food‐web functioning and aquatic ecosystems productivity. These effects are currently overlooked and need to be thoroughly considered in future studies. The use of a mesocosm infrastructure in the present study allowed testing our hypotheses among many different systems in an environmental gradient, but future studies in real aquatic ecosystems are needed. Starting from the knowledge acquired from this study and growing body of research about the plastisphere, future research should investigate the time development of biofouling of different plastic polymers by microalgae investigating at the same time the interaction with other components of aquatic food webs.

## CONFLICT OF INTEREST

The authors declare that there is no conflict of interest.

## Supporting information

Supplementary Material

## Data Availability

The data that support the findings of this study are openly available in Zenodo at http://doi.org/10.5281/zenodo.5674668.
