# Supplementary material for: Microalgae colonization of different microplastic polymers in experimental mesocosms across an environmental gradient
Source: Glob Chang Biol. 2021 Dec 3;28(4):1402–13. doi: 10.1111/gcb.15989 (PMC9299714; doi:10.1111/gcb.15989)
Supplement: Supplementary file 1 — Supplementary Material [file GCB-28-1402-s001.pdf]

Supplementary materials

Microalgae colonization of different microplastic polymers in experimental mesocosms across an environmental gradient

Veronica Nava[1], Miguel Graça Matias[2,3], Andreu Castillo-Escrivà[3], Beata Messyasz[4], Barbara Leoni[1]

[1] Dep. Earth and Environmental Sciences, University of Milano-Bicocca, Piazza della Scienza, 1, 20126, Milano, Italy

[2] Dep. Biogeografía y Cambio Global, Museo Nacional de Ciencias Naturales, CSIC, c/ José Gutiérrez Abascal, 2, 28006 Madrid, Spain

[3] MED - Mediterranean Institute for Agriculture, Environment and Development & Rui Nabeiro Biodiversity Chair, Universidade de Évora. Casa Cordovil 2ª Andar, Rua Dr. Joaquim Henrique da Fonseca, 7000 – 890 Évora, Portugal

[4] Dep. of Hydrobiology, Institute of Environmental Biology, Adam Mickiewicz University in Poznan, Uniwersytetu Poznanskiego 6, 61-614 Poznań, Poland

Figure S1. Raman spectra of the two virgin polymers employed in the study: (a) high-density polyethylene (HDPE); (b) polyethylene terephthalate (PET).

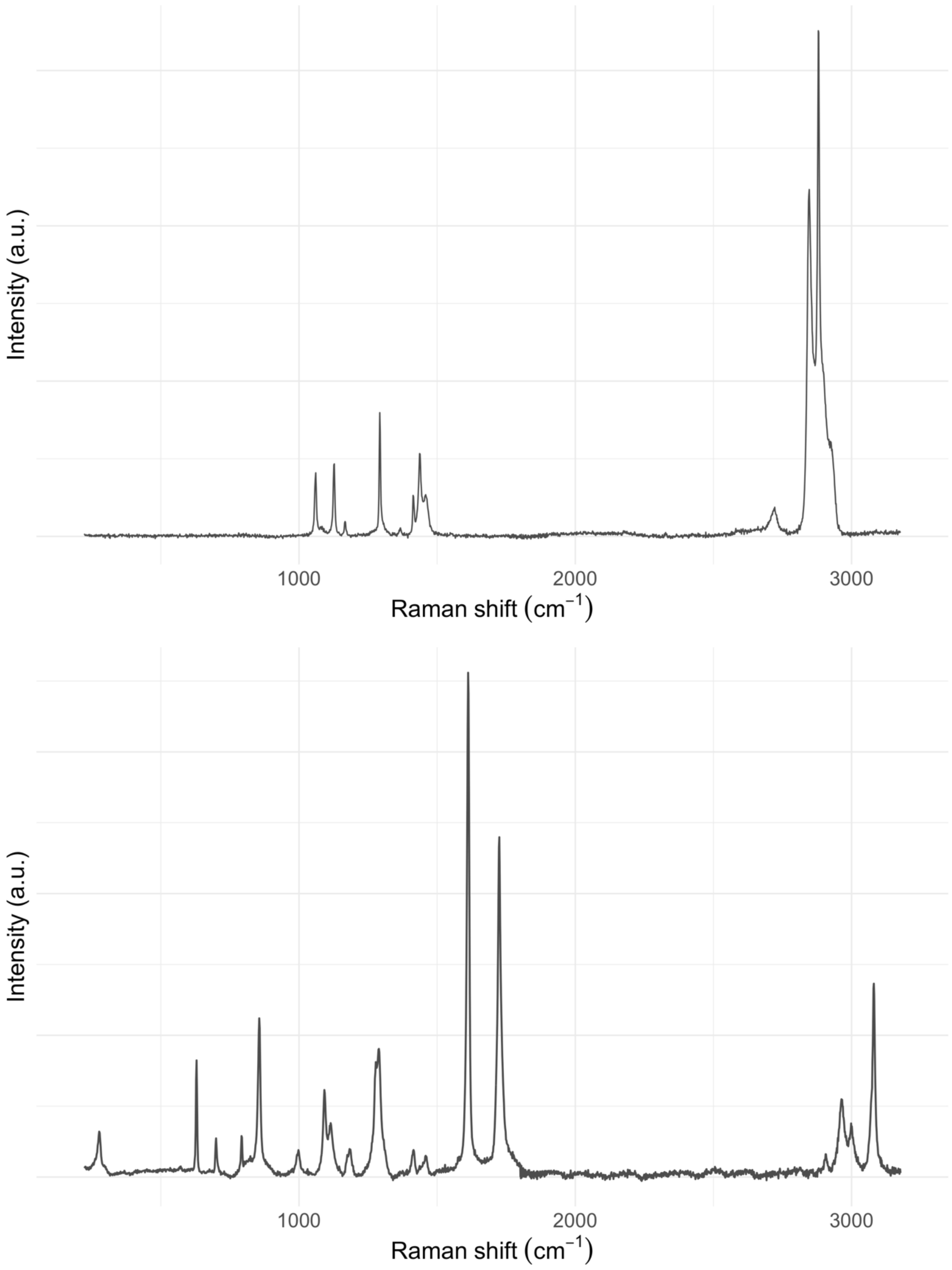

Figure S2. Biomass of microalgae developed on the different plastic polymers in the different mesocosms.

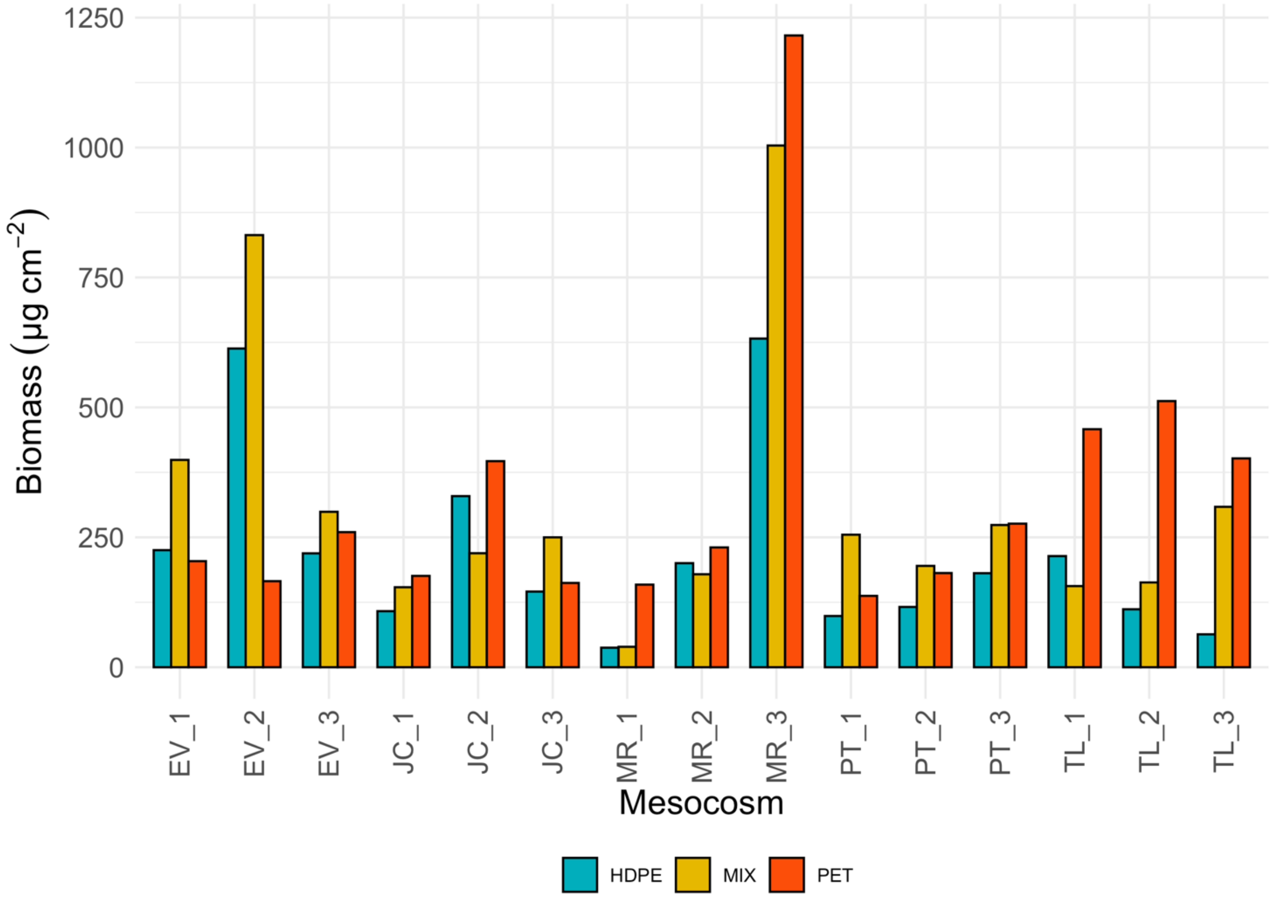

Figure S3. Tukey boxplot of biomass (µg cm^{-2}) of microalgae for different sites on the two plastic polymers and the 'MIX' treatment for the different phyla.

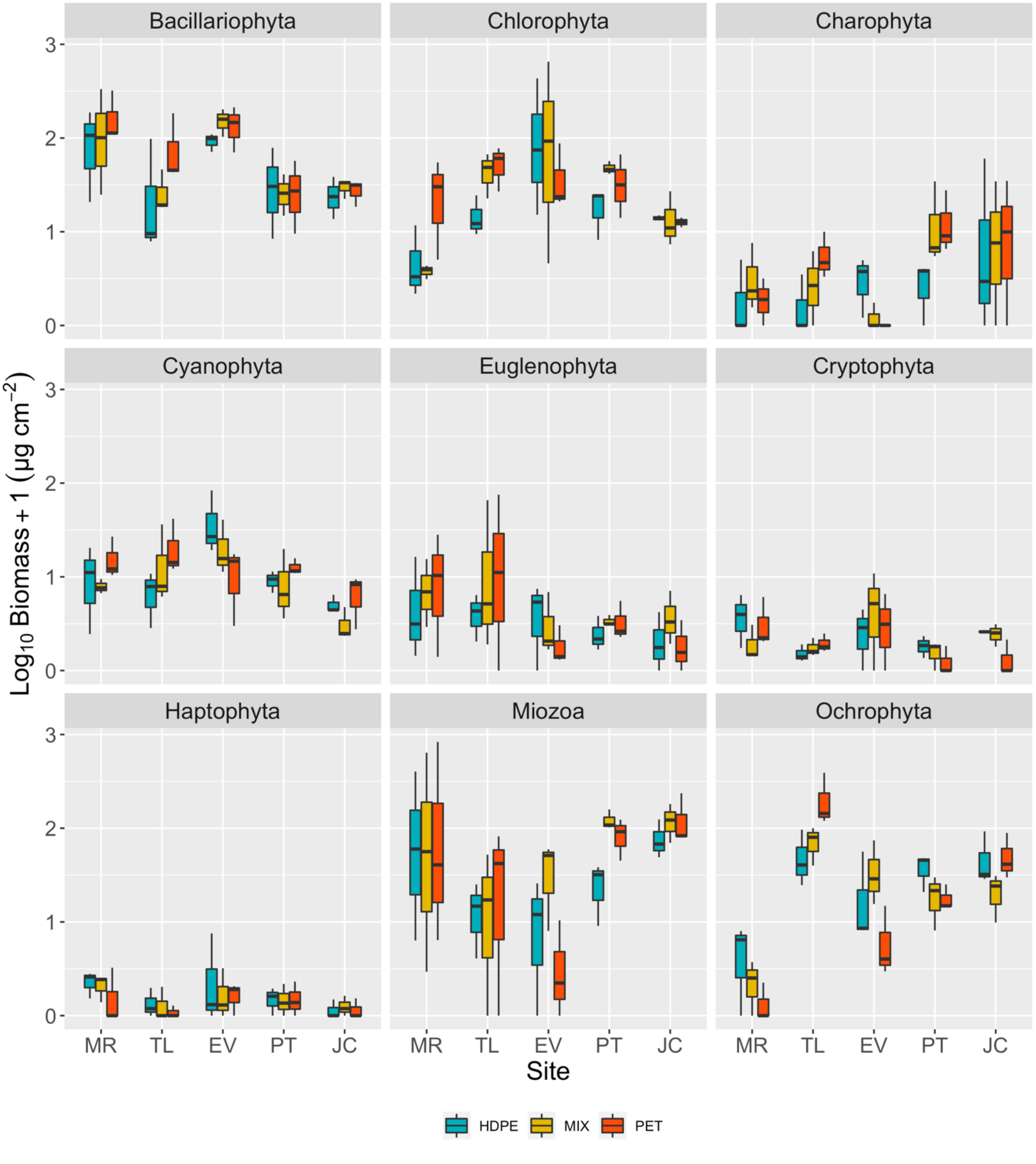

Figure S4. Scatterplot of phosphate concentration (PO_4^-, mg L^{-1}) and total biomass (µg cm^{-2}) for different sites (‘Site’) and different polymer types (‘Polymer’).

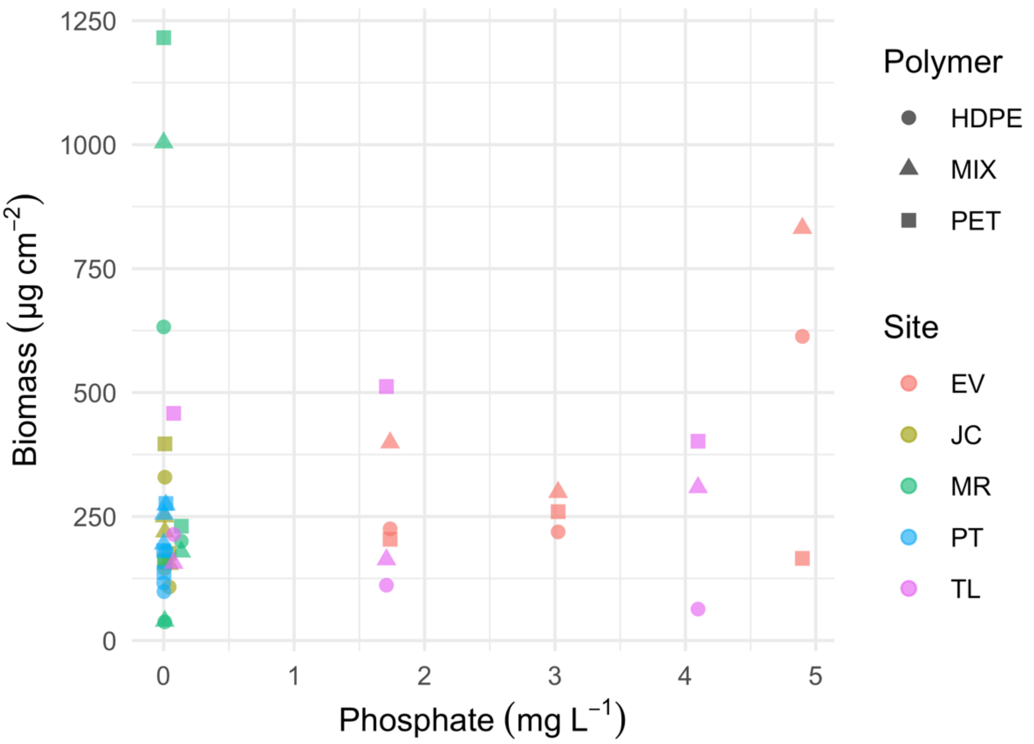

Table S1. Mean air temperature (°C) ± standard error and cumulative rainfall (mm) over the 30 days of duration of the experiments for the different sites.

Site	Temperature (°C)	Rainfall (mm)
Murcia	18.28 ± 0.57	115.20
Toledo	17.03 ± 0.70	64.20
Evora	18.39 ± 0.10	0.00
Porto	17.63 ± 0.60	7.60
Jaca	21.00 ± 0.99	66.40

Table S2. Results of indicator value (IndVal) index for the different sites.

Genus	site	IndVal	*P*-value
Nitzschia	MR	0.7463	0.001***
Pinnularia	MR	0.6426	0.001***
Ulnaria	MR	0.5724	0.001***
Merismopedia	MR	0.5351	0.001***
Chroococcus	MR	0.5300	0.002**
Cocconeis	MR	0.4046	0.004**
Colacium	MR	0.3430	0.047*
Oocystis	TL	0.9381	0.001***
Chromulina	TL	0.7150	0.009**
Jaaginema	TL	0.5256	0.007**
Didymocystis	TL	0.5222	0.003**
Euglena	TL	0.4924	0.031*
Carteria	TL	0.4444	0.003**
Monomorphina	TL	0.3333	0.033*
Navicula	EV	0.8852	0.001***
Caloneis	EV	0.7758	0.001***
Gomphonema	EV	0.7296	0.001***
Staurosira	EV	0.5513	0.001***
Chlamydomonas	EV	0.5305	0.012*
Chamaesiphon	EV	0.4531	0.004**
Scopulonema	EV	0.4448	0.003**
Uroglenopsis	EV	0.3284	0.035*
Platessa	EV	0.3195	0.037*
Aphanocapsa	EV	0.3117	0.041*
Geminella	EV	0.2919	0.033*
Craticula	EV	0.2784	0.041*
Closterium	PT	0.6517	0.001***
Botryococcus	PT	0.4626	0.018**
Peridinium	PT	0.4497	0.013**
Coenocystis	PT	0.4482	0.005**
Synura	PT	0.4212	0.007**
Emergosphaera	PT	0.3976	0.011*
Trachelomonas	PT	0.3454	0.009**
Lyngbya	PT	0.3239	0.025*
Tetraplektron	PT	0.2584	0.044*
Dinobryon	JC	0.7615	0.001***
Tribonema	JC	0.5435	0.002**
Peridiniopsis	JC	0.4200	0.006**
Spirogyra	JC	0.3333	0.031*
Staurastrum	JC	0.3333	0.040*
Mougeotia	JC	0.2858	0.021*

Significance level: * $P < 0.05$; ** $P < 0.01$; *** $P < 0.001$.
